# Supplementary figures and images for: Comparative Analysis of 16S rRNA Gene and Metagenome Sequencing in Pediatric Gut Microbiomes
Source: Front Microbiol. 2021 Jul 15;12:670336. doi: 10.3389/fmicb.2021.670336 (PMC8320171; doi:10.3389/fmicb.2021.670336)

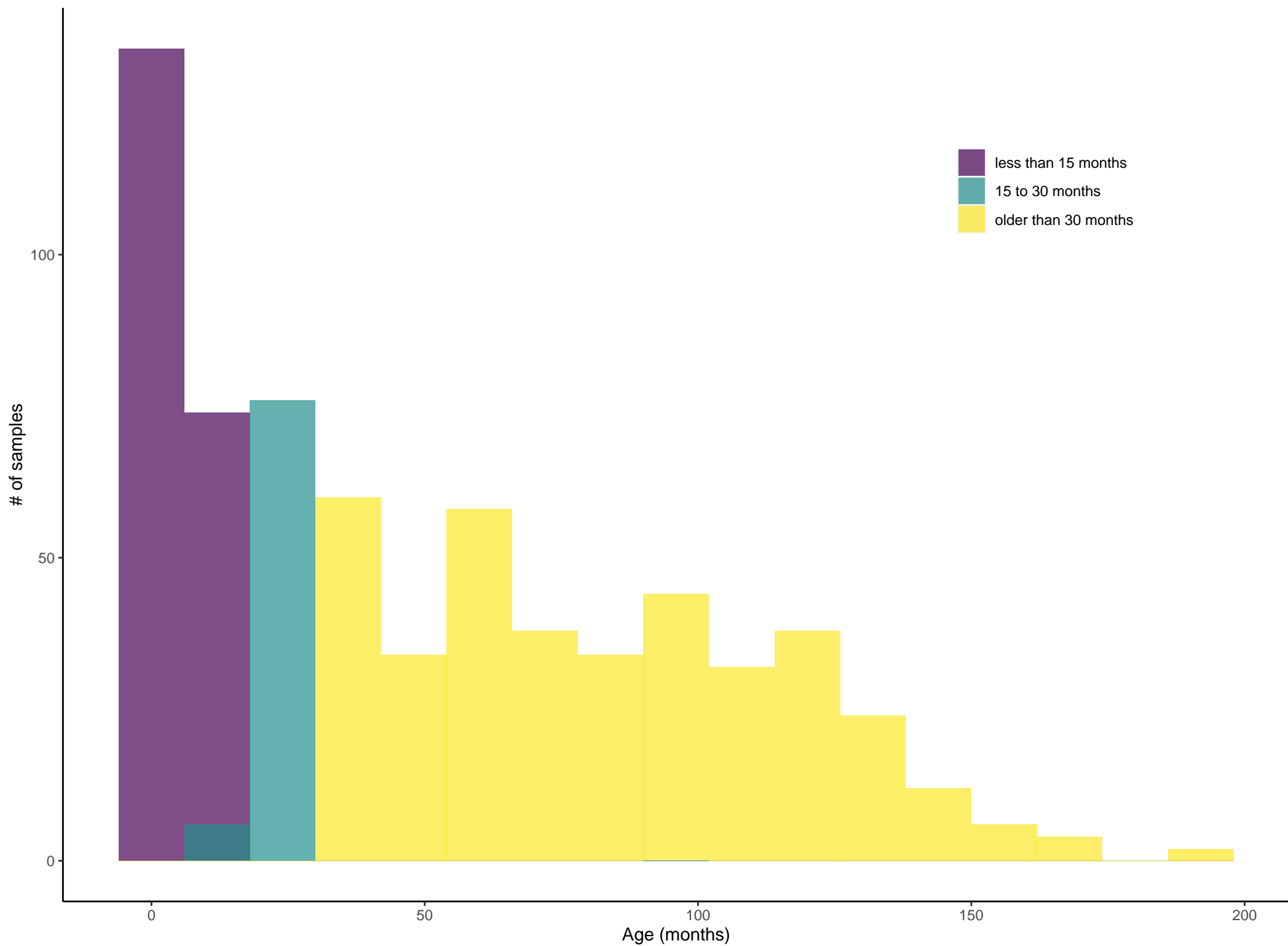

Supplement: Supplementary Figure 1 — RESONANCE: a cohort of healthy children between ages 2 months and 4 years. Histogram showing distribution of ages across developmental stages. Both 16S rRNA gene data and metagenome profiles were obtained for 338 stool samples (one sample per child and timepoint). n = 104 for children <15 months, n = 41 for children 15–30 months, and n = 193 for children >30 months. Color indicates developmental stage. [file Image_1.PDF]

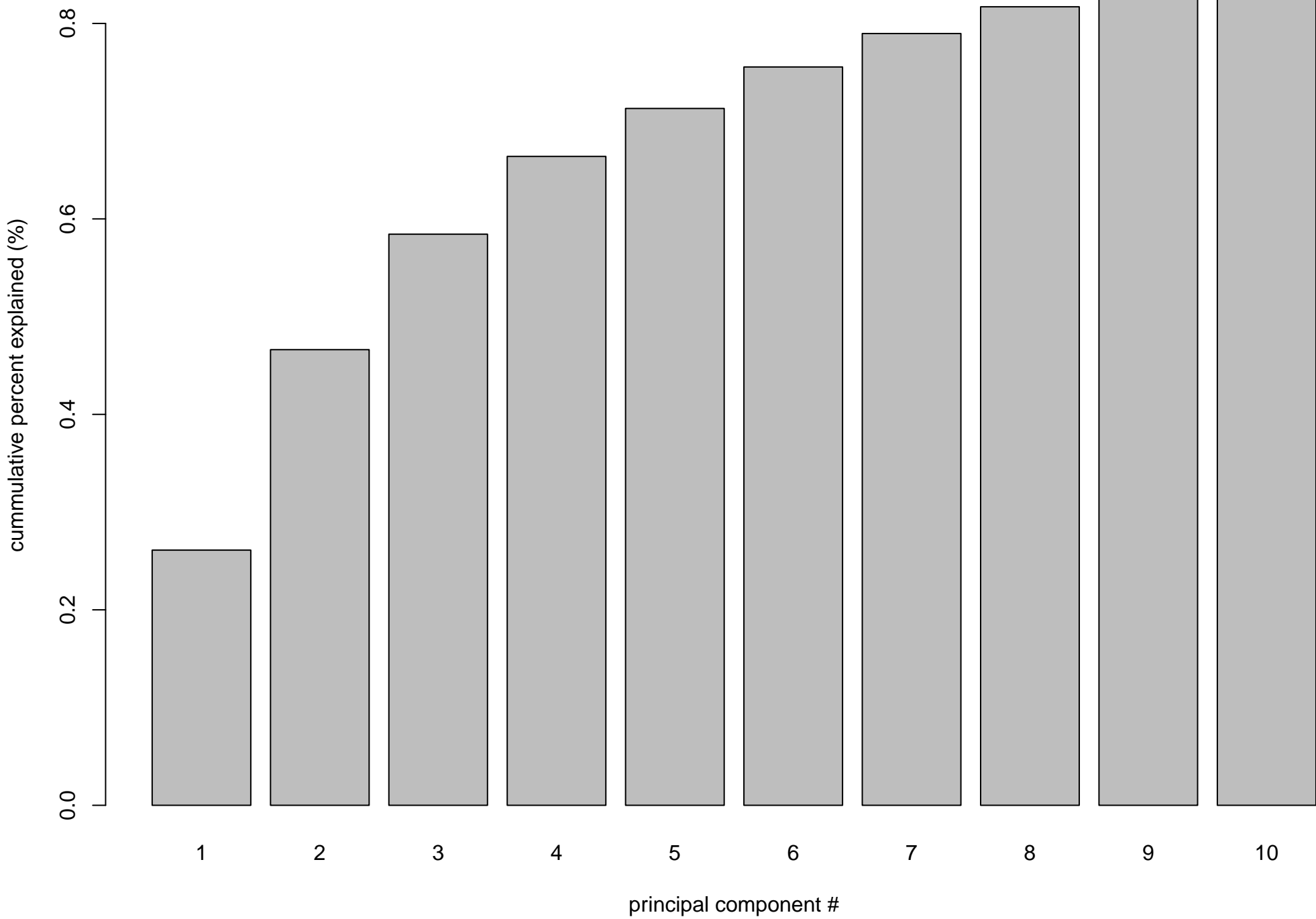

Supplement: Supplementary Figure 2 — Cumulative percent of variation explained by first 100 principal components. Barplot of the cumulative sum of the percentage explained by the first 100 principal components used to create Figure 1D. The first 10 principal components explained 88.55% of the total variation in Bray-Curtis dissimilarity within the dataset. [file Image_2.PDF]

---

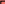 amp  
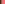 mgx

Supplement: Supplementary Figure 3 — A common phylogenetic tree was generated from all taxa identified by both 16S rRNA gene (amp) and shotgun metagenomic sequencing (mgx). Colors indicate which method was able to identify taxa (peach = identified by 16S, cyan = identified by shotgun metagenomics). Labels at tree tips indicate genera from Figure 2B. [file Image_3.pdf]

A

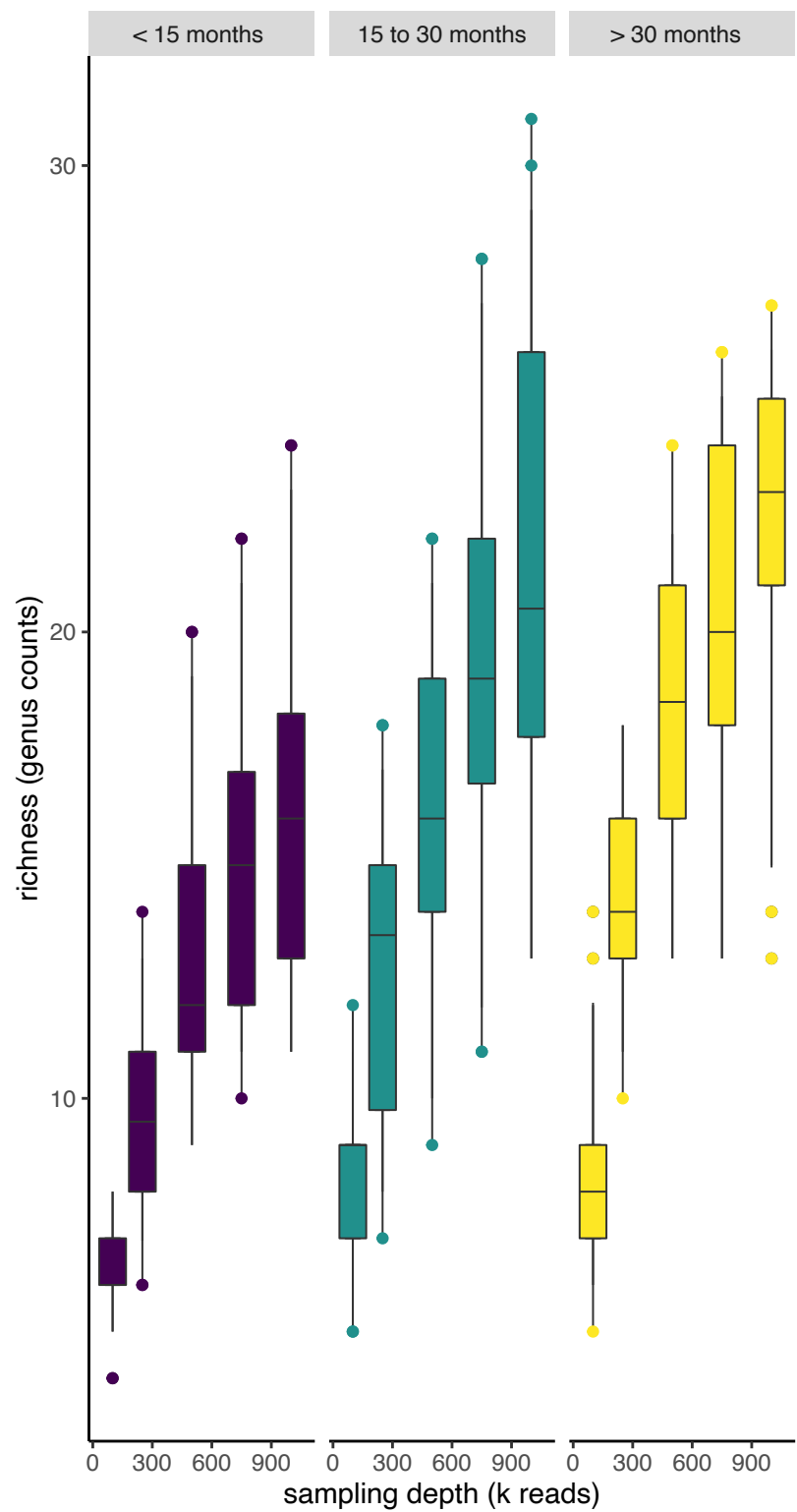

B

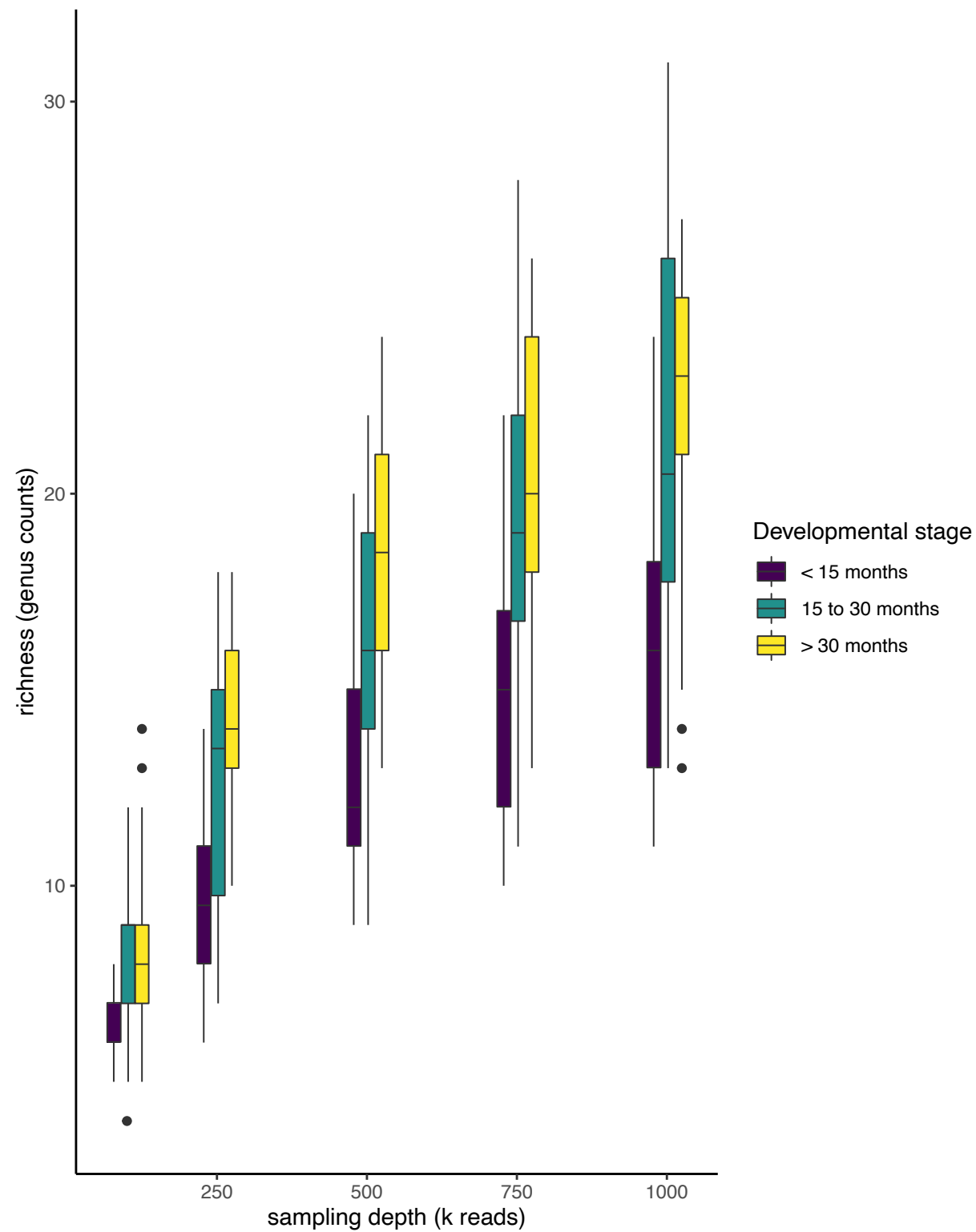

Supplement: Supplementary Figure 4 — Species richness increases with sampling depth within each age group. (A) Boxplots of species richness among all samples at each sampling depth, colored and grouped by developmental stage. (B) Boxplots of species richness among all samples at each re-sampling depth, separated by sampling depth. [file Image_4.pdf]

A

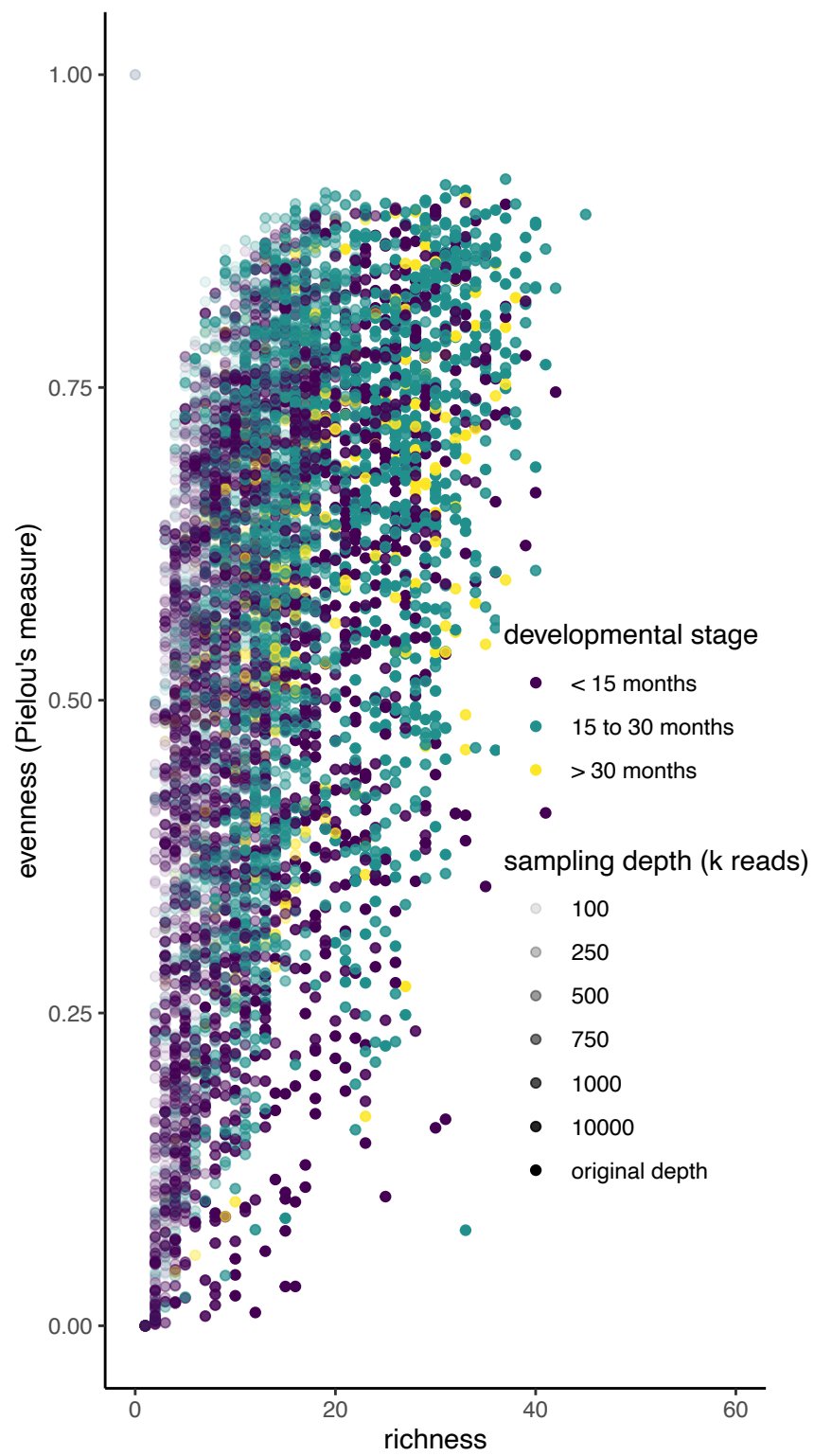

B

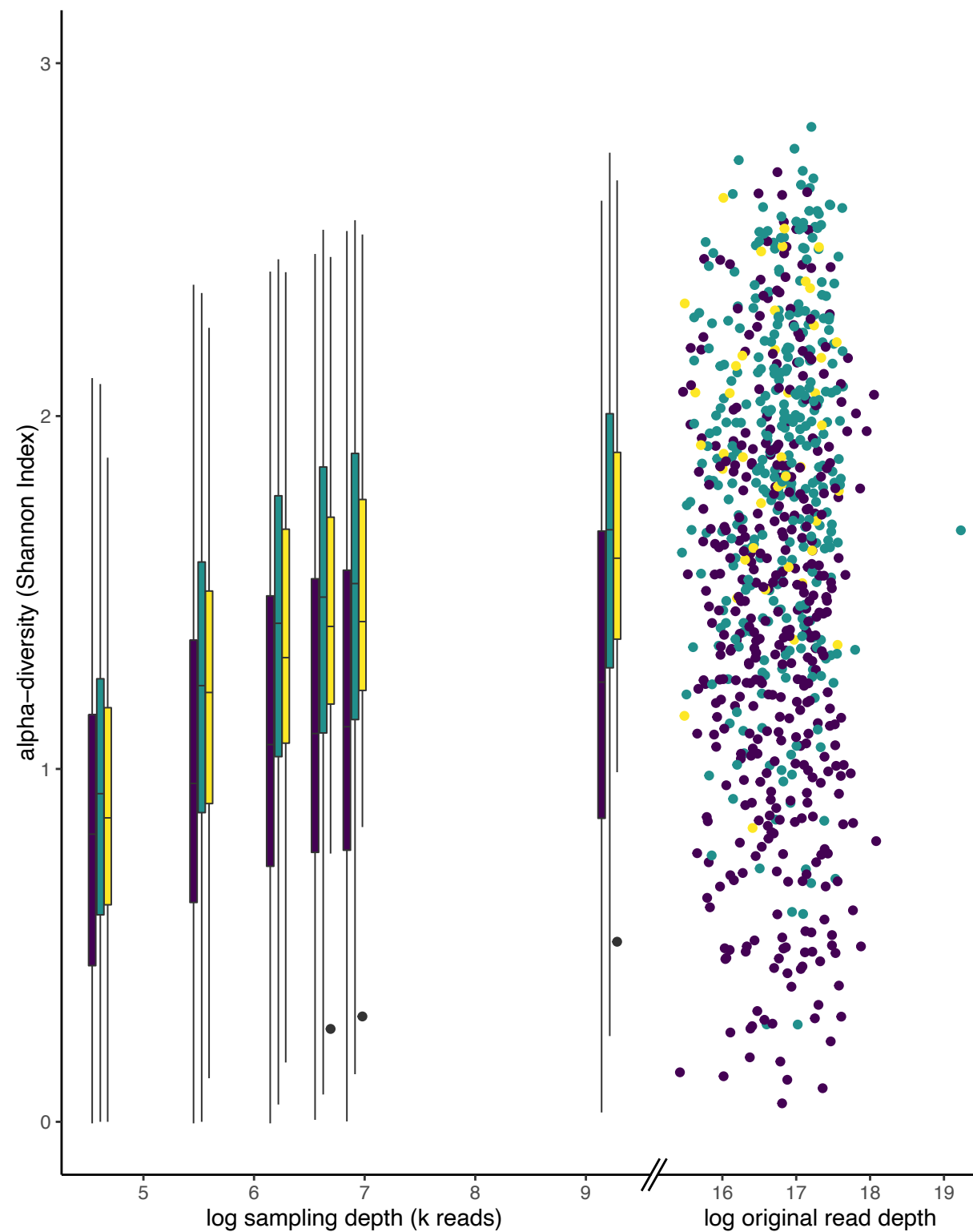

Supplement: Supplementary Figure 5 — Alpha diversity decreases with sequencing depth in DIABIMMUNE dataset. (A) Shotgun metagenomic reads from 804 deeply sequenced samples were resampled times at six different sequencing depths (100k; 250k; 500k; 750k; 1M, and 10M reads). Reads from both cohorts were reassigned taxonomy using MetaPhlAn and diversity was recalculated. Each dot represents a single resampled community. (B) Boxplots of Shannon diversity among all samples at each re-sampling depth, colored by developmental stage. Scatter plot indicates Shannon diversity of original samples. [file Image_5.pdf]

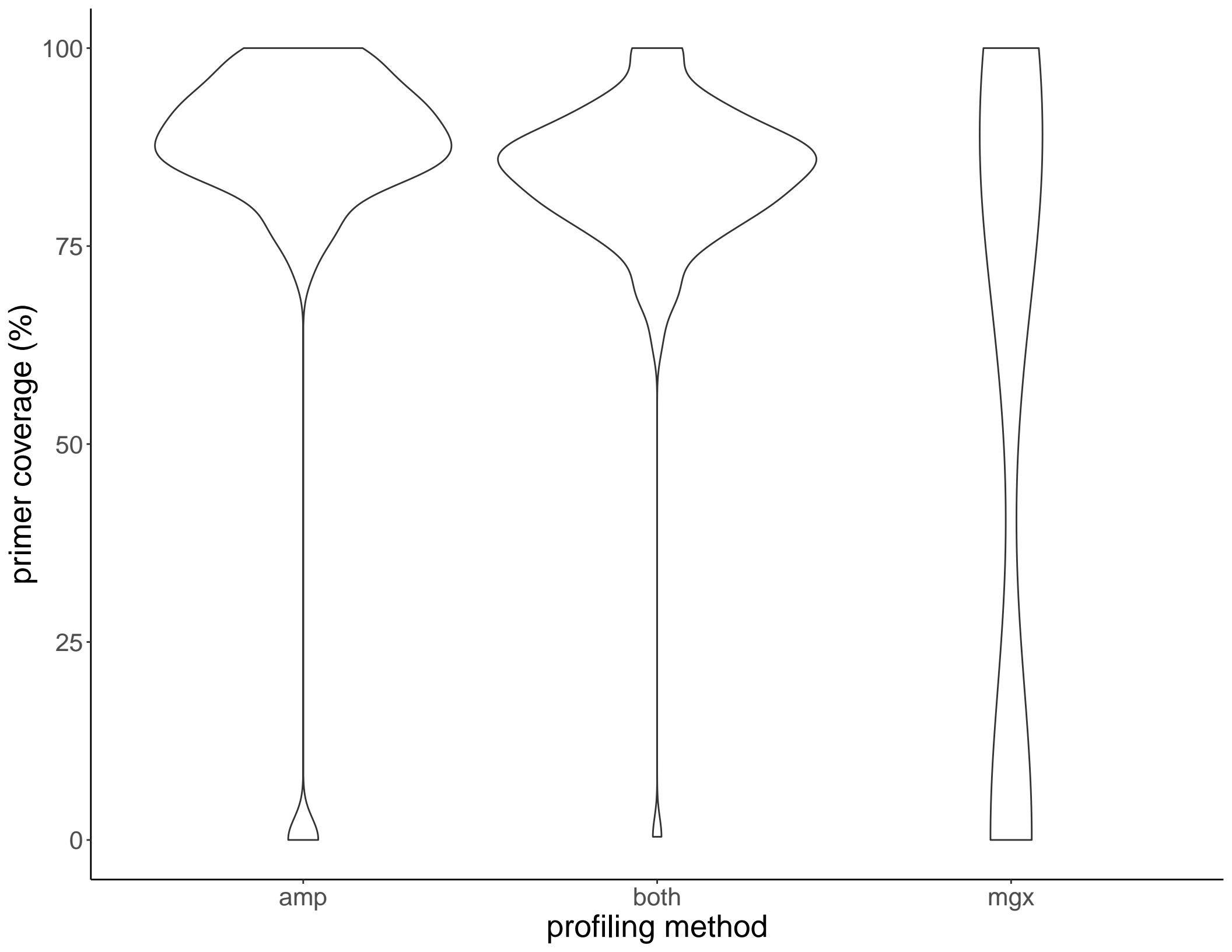

Supplement: Supplementary Figure 6 — Genera found by 16S rRNA amplicon sequencing have significantly higher primer coverage. TestPrime 1.0 was used to calculate the percent primer coverage of the primers used in our study for amplicon sequencing. We compared the percent coverage for microbes found uniquely by 16S rRNA sequencing, both methods, and shotgun metagenomic sequencing. A pairwise Wilcoxon test found that primer coverage for microbes found uniquely by amplicon sequencing is significantly higher than that in the genera found uniquely by shotgun metagenomics (p < 0.05). [file Image_6.pdf]
